# Etymologia: *Picobirnavirus*

**DOI:** 10.3201/eid2601.ET2601

**Published:** 2020-01

**Authors:** Yashpal S. Malik, Souvik Ghosh

**Affiliations:** Indian Veterinary Research Institute, Izatnagar, India (Y.S. Malik);; Ross University School of Veterinary Medicine, Basseterre, St. Kitts, West Indies (S. Ghosh)

**Keywords:** Picobirnavirus, viruses, Picobirnaviridae, double-stranded RNA, genogroups, gastroenteritis, respiratory infections

## *Picobirnavirus* [pi-ko-burґnə-vi″rəs]  

*Picobirnavirus*, the recently recognized sole genus in the family *Picobirnaviridae *(Figure), is a small (Pico, Spanish for small), bisegmented (bi, Latin for two), double-stranded RNA virus. Picobirnaviruses were initially considered to be birna-like viruses, and the name was derived from birnavirus (bisegmented RNA), but the virions are much smaller (diameter 35 nm vs. 65 nm).

**Figure:**
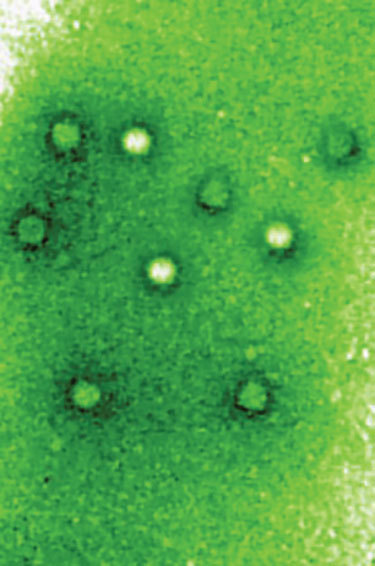
Picobirnavirus by negative stain electron microscopy, from Wikipedia, https://en.wikipedia.org/wiki/File:Picobirnavirus.jpg

Picobirnaviruses are reported in gastroenteric and respiratory infections. These infections were first described in humans and black-footed pigmy rice rats in 1988. Thereafter, these infections have been reported in feces and intestinal contents from a wide variety of mammals with or without diarrhea, and in birds and reptiles worldwide.

## References

[1] Delmas B, Attoui H, Ghosh S, Malik YS, Mundt E, Vakharia VN; Ictv Report Consortium. ICTV virus taxonomy profile: Picobirnaviridae. J Gen Virol. 2019;100:133–4. 10.1099/jgv.0.001186 30484763 PMC12662030

[2] Malik YS, Kumar N, Sharma K, Dhama K, Shabbir MZ, Ganesh B, et al. Epidemiology, phylogeny, and evolution of emerging enteric Picobirnaviruses of animal origin and their relationship to human strains. BioMed Res Int. 2014;2014:780752. 10.1155/2014/780752 25136620 PMC4124650

[3] Pereira HG, Flewett TH, Candeias JA, Barth OM. A virus with a bisegmented double-stranded RNA genome in rat (*Oryzomys nigripes*) intestines. J Gen Virol. 1988;69:2749–54. 10.1099/0022-1317-69-11-2749 3053986

[4] Smits SL, van Leeuwen M, Schapendonk CM, Schürch AC, Bodewes R, Haagmans BL, et al. Picobirnaviruses in the human respiratory tract. Emerg Infect Dis. 2012;18:1539–40. 10.3201/eid1809.120507 22932227 PMC3437736

